# Contribution of Inflammation and Hypoperfusion to White Matter Hyperintensities-Related Cognitive Impairment

**DOI:** 10.3389/fneur.2021.786840

**Published:** 2022-01-04

**Authors:** Chao-Juan Huang, Xia Zhou, Xin Yuan, Wei Zhang, Ming-Xu Li, Meng-Zhe You, Xiao-Qun Zhu, Zhong-Wu Sun

**Affiliations:** Department of Neurology, First Affiliated Hospital of Anhui Medical University, Hefei, China

**Keywords:** cerebral small vessel disease, white matter hyperintensities, lipoprotein-associated phospholipase A2, cerebral blood flow, attention and execution, frontal lobe, thalamus

## Abstract

White matter hyperintensities (WMHs) of presumed vascular origin are one of the most important neuroimaging markers of cerebral small vessel disease (CSVD), which are closely associated with cognitive impairment. The aim of this study was to elucidate the pathogenesis of WMHs from the perspective of inflammation and hypoperfusion mechanisms. A total of 65 patients with WMHs and 65 healthy controls were enrolled in this study. Inflammatory markers measurements [hypersensitive C-reactive protein (hsCRP) and lipoprotein-associated phospholipase A2 (Lp-PLA2)], cognitive evaluation, and pseudocontinuous arterial spin labeling (PCASL) MRI scanning were performed in all the subjects. The multivariate logistic regression analysis showed that Lp-PLA2 was an independent risk factor for WMHs. Cerebral blood flow (CBF) in the whole brain, gray matter (GM), white matter (WM), left orbital medial frontal gyrus [MFG.L (orbital part)], left middle temporal gyrus (MTG.L), and right thalamus (Tha.R) in the patients was lower than those in the controls and CBF in the left triangular inferior frontal gyrus [IFG.L (triangular part)] was higher in the patients than in the controls. There was a significant correlation between Lp-PLA2 levels and CBF in the whole brain (*R* = −0.417, *p* < 0.001) and GM (*R* = −0.278, *p* = 0.025), but not in the WM in the patients. Moreover, CBF in the MFG.L (orbital part) and the Tha.R was, respectively, negatively associated with the trail making test (TMT) and the Stroop color word test (SCWT), suggesting the higher CBF, the better executive function. The CBF in the IFG.L (triangular part) was negatively correlated with attention scores in the Cambridge Cognitive Examination-Chinese Version (CAMCOG-C) subitems (*R* = −0.288, *p* = 0.020). Our results revealed the vascular inflammation roles in WMHs, which may through the regulation of CBF in the whole brain and GM. Additionally, CBF changes in different brain regions may imply a potential role in the modulation of cognitive function in different domains.

## Introduction

Cerebral small vessel disease (CSVD) is a complex cerebrovascular syndrome that comprises diverse clinical, neuropathologic, and neuroimaging manifestations and is associated with cerebrovascular architecture dysfunction (1). The clinical presentations of CSVD are highly heterogeneous, ranging from asymptomatic to vascular cognitive impairment (2), stroke (3), neuropsychiatric symptoms (4), urinary symptoms (5), gait disturbance (6), and so on. The typical pattern of cognitive deterioration involves attention/executive function, information processing speed, and visual space dysfunction (7–9). Neuroimaging markers of CSVD include recent small subcortical infarcts, lacunes, white matter hyperintensities (WMHs) of presumed vascular origin, perivascular spaces, microbleeds, and brain atrophy (10). Among them, WMHs are usually considered the most common neuroimaging manifestation (11) and are defined as diffuse, largely symmetrical WM changes that are hyperintense on T2-weighted sequences, isointense, or hypointense on T1-weighted sequences.

The intricate etiology and pathogenesis of CSVD has been extensively studied in recent study. While age is the most robust determinant of severity and progression of WMHs (12, 13), the complex interaction between hereditary and environmental exposures to hypertension, diabetes mellitus, smoking, and drinking also plays a significant role (14). The underlying mechanisms linked to WMHs include inflammation/oxidative stress (15), ischemia/hypoperfusion (2), barrier/endothelial dysfunction (16), glymphatic system dysfunction (17), and genetic factors. WMHs are related to loss of structural integrity of WM and disruptions in focal to remote functional connectivity and brain networks, which impair the brain reserve and compensatory mechanisms and contribute to worsening clinical outcomes (18). Previous investigations examining systemic and vascular inflammation markers—hypersensitive C-reactive protein (hsCRP) and lipoprotein-associated phospholipase A2 (Lp-PLA2), in the occurrence and development of WMHs, have demonstrated mixed results, with some reporting significant associations (19, 20), yet others finding no correlations (21).

Growing evidence has confirmed a relationship between hypoperfusion and the pathogenesis of WMHs, indicating the likelihood that MRI of the brain plays an essential role not only in diagnosing and characterizing CSVD, but also in quantifying disease burden and exploring potential mechanisms. Arterial spin labeling (ASL) MRI is a clinically available and research technology that uses magnetically labeled arterial blood water protons as endogenous tracers to directly quantify cerebral blood flow (CBF) in a non-invasive manner. ASL displays comparable diagnostic abilities with perfusion gold standard technology—single-photon emission CT (SPECT) and PET (22). The European Consortium in Dementia recommended the implementation of ASL MRI for clinical applications (23). Study has reported that normal-appearing WM (NAWH) regions around WMHs are already in an ischemic state and are more vulnerable to developing into WMHs. The blood flow changes in NAWH may be apparent earlier than the structural changes and visible WMHs (2), so it was speculated that the hemodynamic alterations may be the initiating factor of WMHs. Accordingly, CBF may be a useful neuroimaging marker. To date, relationships between regional CBF and cognitive domains have not been well documented. It was demonstrated that higher baseline CBF of right thalamus and left dorsolateral prefrontal cortex was related to better executive function (24). Moreover, a longitudinal study found that higher baseline CBF of frontal lobe was linked to better episodic memory and that lower CBF of cingulate gyrus was related to more rapid cognitive impairment (25).

The purpose of this study was to determine the mechanisms of inflammation and hypoperfusion in the pathogenesis and cognitive impairment of WMHs. To date, studies examining WMHs from the perspective of multiple mechanisms are lacking. This study can help to target potentially modifiable biomarkers for prevention and intervention therapies to maintain cognition in patients with WMHs.

## Materials and Methods

### Subjects

A total of 65 right-handed consecutive patients with WMHs (aged 50 to 80 years) were enrolled from the First Affiliated Hospital of Anhui Medical University. Patients with WMHs were diagnosed as presenting the Fazekas score of 2–3 for either deep or periventricular WMHs on fluid-attenuated inversion recovery (FLAIR) images, according to previous criterion (26). These patients were initially recruited with slight symptoms such as dizziness, headache, or cognitive decline. The exclusion criteria were as follows: (1) history of brain tumor, traumatic injury, or intracranial operation; (2) cerebral hemorrhage, acute cerebral infarction, or cerebrovascular stenosis; (3) WMHs from nonvascular diseases such as poisoning, multiple sclerosis, encephalitis, or infection; (4) history of aging-related diseases that cause cognitive impairment such as Parkinson's disease, Alzheimer's disease (AD), or Lewy body dementia; (5) severe visuospatial deficits, hearing impairments, or language disorders; (6) major neurological or psychiatric illness or drug/alcohol use disorder; (7) *in-vivo* dentures or metallic stents; (8) liver, kidney, heart, and lung dysfunction or systemic malignant tumors; and (9) acute or chronic inflammatory diseases.

A total of 65 healthy controls (HCs) matched by age, sex, and education were enrolled in this study at the same time. This cross-sectional study was approved by the Ethics Committee of the First Affiliated Hospital of Anhui Medical University and a written informed consent was obtained from all the participants.

### Neuropsychological Assessment

A series of neuropsychological assessments was performed by 2 experienced neurologists within 1 week of MRI scanning. The Cambridge Cognitive Examination–Chinese Version (CAMCOG-C), Mini-Mental State Examination (MMSE), Montreal Cognitive Assessment (MOCA), Stroop color word test (SCWT) (SCWT-A: dot; SCWT-B: word; SCWT-C: color word), and trail making test (TMT) (TMT-A; TMT-B) were applied to evaluate all the cognitive domains–global cognition, episodic memory, attention, executive function, psychomotor speed, and visuospatial function. The neuropsychological assessments took approximately 2 h.

### Laboratory Biomarker Measurement

Alanine transaminase (ALT), aspartate transaminase (AST), blood urea nitrogen (BUN), creatinine (CR), uric acid (UA), total cholesterol (TCH), triglyceride (TG), high-density lipoprotein (HDL), glucose, hsCRP, and Lp-PLA2 were measured within 1 week of MRI scanning. Fasting blood samples were collected in tubes containing Ethylenediaminetetraacetic acid (EDTA) and centrifuged at 3,500 rpm for 8 min and aliquots of plasma were stored at −40°C until used for biochemical analyses. Plasma Lp-PLA2 was detected with a double-antibody sandwich ELISA kit (Kangerke Biotech Corporation Ltd., Tianjin, China).

### Magnetic Resonance Imaging Acquisition

The GE 3.0 Tesla MR System (Discovery MR750w, Milwaukee, Wisconsin, USA) with a 24-channel head coil was used in this study. All the participants were instructed to keep their eyes closed without falling asleep and to think as little as possible during the scan. High-resolution T1-weighted images were collected with 188 sagittal slices covering the whole brain. The scan parameters were as follows: slice thickness = 1.0 mm, repetition time (TR) = 8.464 ms, echo time (TE) = 3.248 ms, flip angle (FA) = 12°, field of view (FOV) = 256 mm^2^ × 256 mm^2^, matrix size = 256 × 256, and acquisition time = 4 min 56 s. T2 FLAIR images were acquired with following parameters: TR = 9,000 ms, TE = 119.84 ms, FA = 160°, FOV = 225 mm^2^ × 225 mm^2^, matrix size = 512 × 512, 19 contiguous slices with thickness of 7.0 mm, and scan time = 1 min 57 s. Pseudocontinuous ASL (PCASL) parameters were as follows: TR = 5,070 ms, TE = 11.48 ms, FA = 111°, FOV = 240 mm^2^ × 240 mm^2^, matrix size = 128 × 128, slice thickness = 3.0 mm, with postlabeling delay time = 2,000 ms, label duration = 1,500 ms, 50 slices covering the whole brain, and acquisition time = 4 min 54 s. Susceptibility weighted imaging (SWI) parameters were as follows: TR = 45.4 ms, TE = 23.536 ms, FA = 20°, FOV = 240.64 mm^2^ × 240.64 mm^2^, matrix size = 512 × 512, 138 contiguous slices with thickness of 1.0 mm, and scan time = 3 min 51 s.

### Image Processing and Analysis

Cerebral blood flow maps were generated by the GE server automatically. An PCASL difference image was calculated after subtraction of the label image from the control image. The 3 PCASL difference images were averaged to calculate the CBF maps in combination with the proton density-weighted reference images. The detailed calculation procedures have been described in a previous study (27). Then, the MATrix LABoratory (MATLAB)-based Statistical Parametric Mapping V8 (SPM8) (https://www.fil.ion.ucl.ac.uk/spm/software/spm8/) software package was used to analyze the CBF data obtained from the server. A one-step normalization of the CBF map was conducted: the Montreal Neurological Institute (MNI) PET template image to match the CBF map was specified. To minimize individual differences, the CBF map was processed with the Z-standardization method (CBF of each voxel minus the mean CBF of whole brain and then divided by the SD) to achieve standardization. After standardization, the CBF map was smoothed with a Gaussian kernel of 6 mm Full-Width at Half-Maximum (FWHM). Multiple comparisons were corrected using the cluster-level false discovery rate (FDR) method, resulting in a cluster defining threshold of *p* < 0.001 and a corrected cluster significance of *p* < 0.05 (28). Clusters were localized by non-linear transformation to the anatomical automatic labeling (AAL) template provided by the Montreal Institute of Neuroscience. The results are presented using xjView (http://www.alivelearn.net/xjview/ad.php).

### Statistical Analysis

The Shapiro–Wilk (S-W) test was performed on all the measures to assess data normality. Two-sample two-tailed *t*-tests were used for normally distributed continuous variables and the Wilcoxon's rank-sum test were used for non-normally distributed numerical variables. The categorical variables comparison was performed by the chi-squared tests. The multivariate logistic regression analysis was used to investigate the independent risk factors for WMHs, taking variables with statistical differences and clinical significance as input variables. The Pearson's correlation analysis and the Spearman's correlation analysis were applied to explore the relationship between normally distributed data and non-normally distributed data, respectively. All the statistical analyses were performed using the SPSS version 25.0 with significance set at *p* < 0.05.

## Results

### Demographic, Clinical, and Neuroimaging Manifestations Results

A total of 130 subjects were recruited including 65 patients with WMHs and 65 HCs. The demographic, clinical, and neuroimaging manifestations results are shown in Table 1. We noted significant differences in the prevalence of hypertension (*p* < 0.001), ever drinking history (*p* = 0.046), and Lp-PLA2 levels (*p* = 0.022) between patients with WMHs and HCs. However, no significant differences in sex, age, education, history of diabetes, hyperlipidemia, operation, and smoking were found between the two groups. There were also no significant differences in UA, TCH, TG, HDL, GLU, and hsCRP levels. Compared with the HCs, patients with WMHs have impaired cognitive functions in domains of total cognitive, attention, and executive function. Besides, there were 26 patients coexisted with lacunes and 19 patients coexisted with microbleeds. The CBF values in the whole brain, gray matter (GM), and WM were significantly lower in the WMHs group than those of HCs.

**Table 1:** Demographic, clinical, and neuroimaging manifestations data.

	**HCs (*n =* 65)**	**patients with WMHs (*n =* 65)**	***P* Value**
Female/Male	32/33	29/36	0.598^a^
Age (years)	58.00 (55.00,64.50)	63.00 (56.00,67.50)	0.076^b^
Education (years)	9.20 ± 3.65	8.63 ± 3.89	0.391^c^
Hypertension (*n*, %)	9 (13.85)	27 (41.54)	**<0.001** ^a^
Diabetes (*n*, %)	3 (4.62)	9 (13.85)	0.069^a^
Hyperlipidemia (*n*, %)	7 (10.77)	13 (20.00)	0.145^a^
Operation (*n*, %)	31 (47.69)	26 (40.00)	0.377^a^
Smoking (*n*, %)	12 (18.46)	17 (26.15)	0.292^a^
Drinking (*n*, %)	12 (18.46)	22 (33.85)	**0.046** ^a^
UA (μmol/L)	316.00	327.00	0.470^b^
	(266.50,374.00)	(280.00,371.00)	
TCH (mmol/L)	4.67 ± 0.99	4.73 ± 1.02	0.718^c^
TG (mmol/L)	1.33 (0.92,2.07)	1.27 (0.86,1.95)	0.790^b^
HDL (mmol/L)	1.43 (1.13,1.71)	1.33 (1.16,1.55)	0.457^b^
GLU (mmol/L)	5.76 (5.38,6.31)	5.65 (5.13,6.25)	0.308^b^
HsCRP (mg/L)	0.90 (0.50,1.60)	1.07 (0.60,2.28)	0.083^b^
Lp-PLA2 (ng/ml)	108.64 ± 37.97	124.08 ± 38.14	**0.022** ^c^
CAMCOG-C	92.00 (88.50,96.00)	84.00 (75.00,93.00)	**<0.001** ^b^
Orientation	10.00 (10.00,10.00)	10.0 (9.00,10.00)	**0.002** ^b^
Language	27.00 (26.00,28.00)	26.00 (23.00,28.00)	**<0.001** ^b^
Memory	20.00 (18.00,22.00)	18.00 (14.00,21.00)	**0.003** ^b^
Attention	7.00 (6.00,7.00)	6.00 (5.00,7.00)	**0.003** ^b^
Execution	12.00 (11.00,12.00)	11.00 (8.50,12.00)	**0.005** ^b^
Calculation	2.00 (2.00,2.00)	2.00 (2.00,2.00)	**0.004** ^b^
Abstraction	6.00 (6.00,8.00)	5.00 (4.00,7.00)	**0.002** ^b^
Perception	8.00 (7.50,9.00)	7.00 (6.00,8.00)	**<0.001** ^b^
MMSE	29.00 (27.00,30.00)	27.00 (22.00,29.00)	**<0.001** ^b^
MOCA	25.00 (23.00,27.00)	22.00 (17.00,25.50)	**<0.001** ^b^
SCWT A (dot)	18.48 (14.69,23.14)	25.78 (18.57,35.40)	**<0.001** ^b^
SCWT B (word)	22.88 (18.12,26.05)	28.16 (22.16,35.59)	**<0.001** ^b^
SCWT C (color word)	33.21 (27.54,41.26)	38.46 (31.14,47.24)	**0.005** ^b^
TMT-A	55.87 (42.19,70.00)	77.76 (59.85,154.10)	**<0.001** ^b^
TMT-B	96.88 (74.17,132.50)	159.47 (106.31,300.00)	**<0.001** ^b^
Coexist with lacunes	0 (0.00)	26 (40.00)	**<0.001** ^a^
Coexist with	0 (0.00)	19 (29.23)	**<0.001** ^a^
microbleeds			
Whole brain CBF	0.019 ± 0.005	0.017 ± 0.005	**0.029** ^c^
GM CBF	0.128 ± 0.041	0.103 ± 0.040	**<0.001** ^c^
WM CBF	−0.147 ± 0.096	−0.185 ± 0.095	**0.026** ^c^

a *χ^2^ value of the chi-squared test*;

b *Z value of Wilcoxon's rank-sum test*;

c *T value of t-test*.

*Significant differences are indicated in bold. HCs, healthy controls; WMHs, white matter hyperintensities; UA, uric acid; TCH, total cholesterol; TG, triglyceride; HDL, high density lipoprotein; GLU, glucose; hsCRP, hypersensitive C-reactive protein; Lp-PLA2, lipoprotein-associated phospholipase A2; CAMCOG-C, Cambridge Cognitive Examination-Chinese Version; MMSE, Mini-Mental State Examination; MOCA, Montreal Cognitive Assessment; SCWT, Stroop color word test; TMT, trail making test; GM, gray matter; WM, white matter; CBF, cerebral blood flow*.

### Logistic Regression Analysis of the Risk Factors for Patients With WMHs

To further evaluate the correlated risk factors for WMHs, we established a logistic regression model (Table 2) for the factors that may affect WMHs. The results indicated that only hypertension (*B* = 1.779, *p* = 0.001) and Lp-PLA2 level (*B* = 0.013, *p* = 0.028) could significantly affect the appearance of WMHs after adjusting for confounding variables.

**Table 2:** The results of the logistic regression analysis of clinical risk factors for WMHs.

	**B**	**SE**	**Walds**	***P***	***OR (95%CI)***
Sex (Male)	0.191	0.543	0.124	0.724	1.211 (0.418–3.508)
Age	0.044	0.032	1.940	0.164	1.045 (0.982–1.111)
Education	−0.100	0.064	2.462	0.117	0.905 (0.799–1.025)
Hypertension	1.779	0.534	11.106	**0.001**	5.925 (2.081–16.869)
Diabetes	1.920	0.987	3.785	0.052	6.818 (0.986–47.148)
Hyperlipidemia	0.082	0.689	0.014	0.905	1.086 (0.281–4.190)
Smoking	0.218	0.635	0.118	0.731	1.244 (0.358–4.319)
Drinking	0.428	0.571	0.562	0.454	1.534 (0.501–4.700)
TCH	0.169	0.215	0.621	0.431	1.184 (0.778–1.804)
TG	−0.227	0.233	0.949	0.330	0.797 (0.505–1.258)
GLU	−0.199	0.219	0.824	0.364	0.820 (0.534–1.259)
hsCRP	0.175	0.117	2.242	0.134	1.191 (0.947–1.496)
LP–PLA2	0.013	0.006	4.824	**0.028**	1.013 (1.001–1.024)

*Significant differences are indicated in bold. WMHs, white matter hyperintensities; B, regression coefficient; Walds, Wald test; OR, odds ratio; TCH, total cholesterol; TG, triglyceride; GLU, glucose; hsCRP, hypersensitive C-reactive protein; Lp-PLA2, lipoprotein-associated phospholipase A2*.

### Group Comparison of the CBF

**Figure 1:**
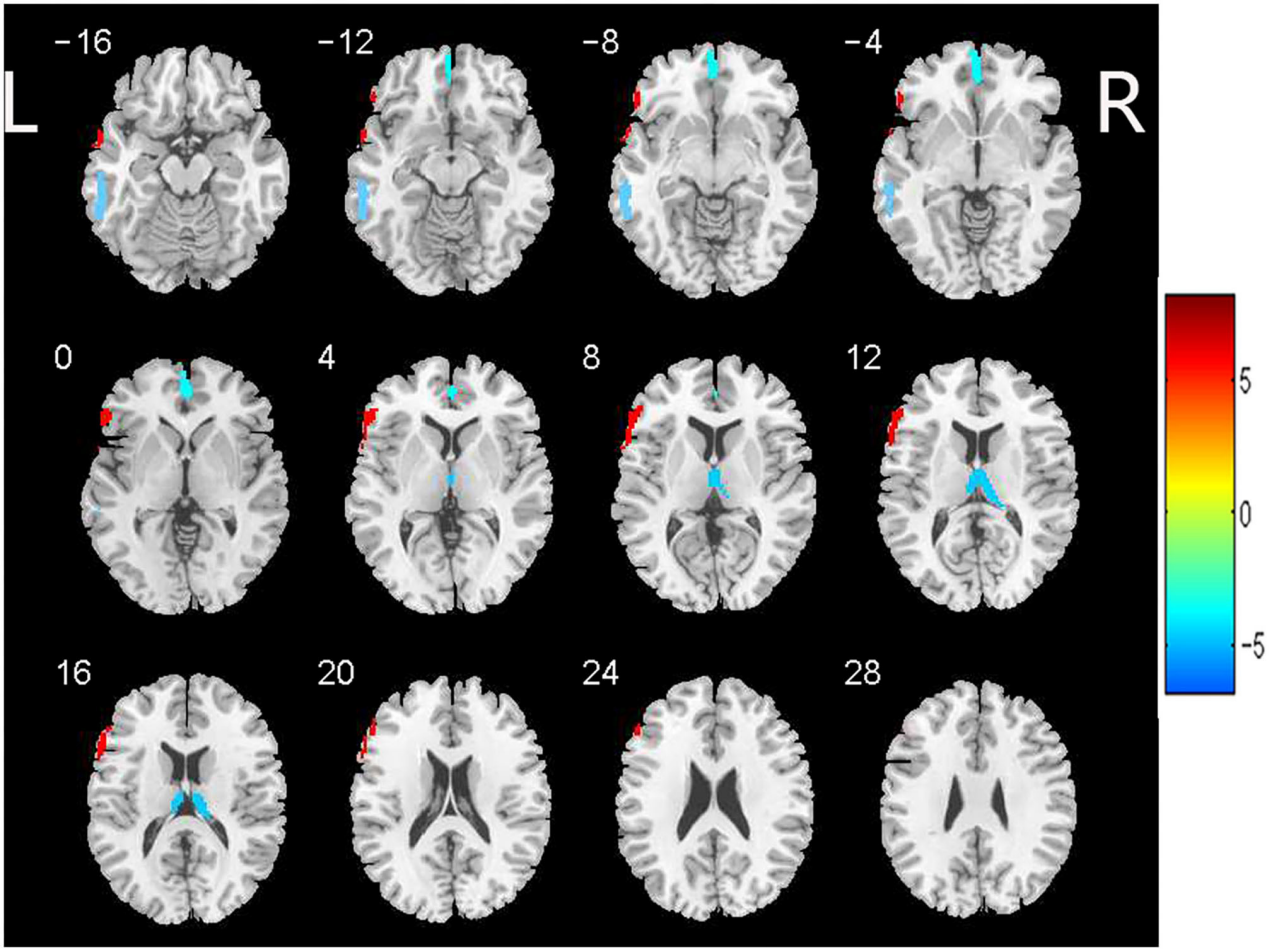
Brain regions with significant differences in CBF between patients with WMHs and HCs. Significant decreased CBF in the left orbital medial frontal gyrus [MFG.L (orbital part)], left middle temporal gyrus (MTG.L) and right thalamus (Tha.R) was observed in the patients with WMHs relative to the HCs. While increased CBF was found in the left triangular inferior frontal gyrus [IFG.L (triangular part)] in WMHs group compared to the HCs. These regions were selected as regions of interest. The color bar indicates the *T* score. CBF, cerebral blood flow; WMHs, white matter hyperintensities; HCs, healthy controls.

Based on the analysis of CBF data, 4 large clusters were identified through cluster-level FDR method as shown in Table 3 and Figure 1. The CBF values in the left orbital medial frontal gyrus [MFG.L (orbital part)], left middle temporal gyrus (MTG.L) and right thalamus (Tha.R) were significantly lower in the WMHs group than those of the HCs. Notably, in the left triangular inferior frontal gyrus [IFG.L (triangular part)], the CBF values in the WMHs group were significantly higher than those from the HCs. These regions with differing perfusion were extracted as regions of interest (ROIs) for further correlation analysis.

**Table 3:** Brain regions with significant differences in CBF between patients with WMHs and HCs.

**Brain regions (AAL)**	**Cluster size** **(voxels)**	**Peak voxel coordinate—** **MNI coordinates**			***T*-value**
		**x**	**y**	**z**	
**Decreased regions**					
MFG.L (orbital part)	236	−2	62	−6	−4.223
MTG.L	499	−58	−26	−24	−4.763
Tha.R	289	10	−18	16	−5.374
**Increased regions**					
IFG.L (triangular part)	565	−58	32	2	5.440

*WMHs, white matter hyperintensities; HCs, healthy controls; AAL, anatomical automatic labeling; MNI, Montreal Neurological Institute; MFG.L (orbital part), left orbital medial frontal gyrus; MTG.L, left middle temporal gyrus; Tha.R, right thalamus; IFG.L (triangular part), left triangular inferior frontal gyrus*.

### Association Between Lp-PLA2 and Whole Brain, GM, and WM CBF

**Figure 2:**
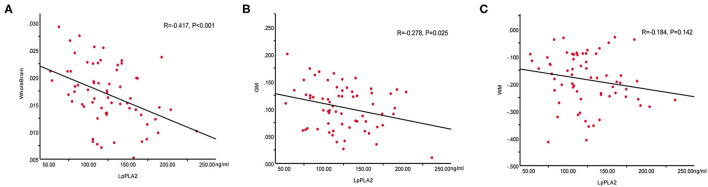
Correlations between plasma Lp-PLA2 levels and CBF values in the whole brain, GM and WM in patients with WMHs. **(A)** Negative correlation between Lp-PLA2 levels and whole brain CBF. **(B)** Negative correlation between Lp-PLA2 levels and GM CBF. **(C)** No correlation between Lp-PLA2 levels and WM CBF. Lp-PLA2, lipoprotein-associated phospholipase A2; CBF, cerebral blood flow; GM, gray matter; WM, white matter; WMHs, white matter hyperintensities.

To evaluate whether there was a relationship between Lp-PLA2 levels and CBF values in patients with WMHs, the Pearson's correlation analysis between plasma Lp-PLA2 levels and CBF values was analyzed. As shown in Figure 2, there were significant negative correlations between Lp-PLA2 levels and CBF in the whole brain (*R* = −0.417, *p* < 0.001) and GM (*R* = −0.278, *p* = 0.025) in the patients. However, there was no significant correlation between Lp-PLA2 levels and CBF in WM (*R* = −0.184, *p* = 0.142).

### Relationship Between CBF in ROIs and Neuropsychological Tests

**Figure 3:**
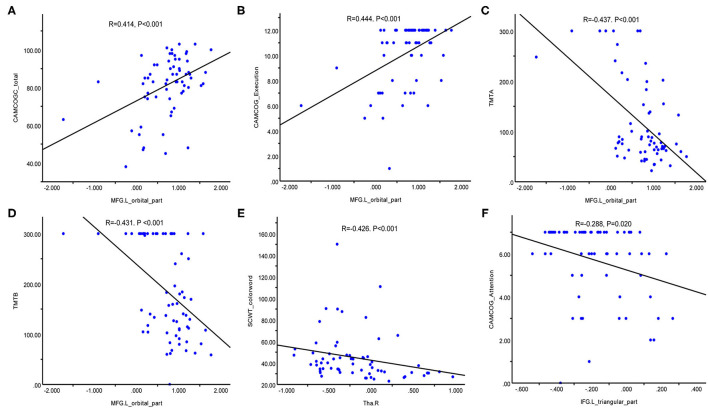
Correlation between CBF in regions of interest and neuropsychological test results in patients with WMHs. **(A)** Positive correlation between MFG.L (orbital part) CBF values and the CAMCOG-C total scores. **(B)** Positive correlation between MFG.L (orbital part) CBF and execution based on the CAMCOG-C scores. **(C)** Negative correlation between MFG.L (orbital part) CBF values and TMT-A scores. **(D)** Negative correlation between MFG.L (orbital part) CBF values and TMT-B scores. **(E)** Negative correlation between Tha.R CBF values and SCWT-C scores. **(F)** Negative correlation between IFG.L (triangular part) CBF values and attention based on the CAMCOG-C scores. CBF, cerebral blood flow; MFG.L (orbital part), left orbital medial frontal gyrus; Tha.R, right thalamus; IFG.L (triangular part), left triangular inferior frontal gyrus; CAMCOG-C, Cambridge Cognitive Examination-Chinese Version; TMT, trail making test; SCWT, Stroop color word test.

Figure 3 vividly illustrates the correlations between CBF values in ROIs and neuropsychological tests in patients with WMHs. The CBF of MFG.L (orbital part) was positively correlated with total scores of the CAMCOG-C scores (*R* = 0.414, *p* < 0.001) (Figure 3A) and execution based on the CAMCOG-C scores (*R* = 0.444, *p* < 0.001) (Figure 3B) and negatively correlated with TMT-A (*R* = −0.437, *p* < 0.001) (Figure 3C) and TMT-B scores (*R* = −0.431, *p* < 0.001) (Figure 3D). The CBF of Tha.R was negatively correlated with SCWT-C scores (*R* = −0.426, *p* < 0.001) (Figure 3E). Moreover, CBF of IFG.L (triangular part) was also negatively correlated with attention based on the CAMCOG-C scores (*R* = −0.288, *p* = 0.020) (Figure 3F). However, there was no significant correlation between CBF of MTG.L and neuropsychological test results. The other correlation results with respect to perfusion and cognitive are given in Supplementary Table 1 and Supplementary Figure 1.

## Discussion

To date, the exact mechanism of WMHs remains unclear and studies examining WMHs from the perspective of multiple mechanisms are still lacking. This study was to determine the effects of inflammation and hypoperfusion in the pathogenesis and cognitive impairment of WMHs. We found that Lp-PLA2 could be seemed as an independent risk factor for WMHs. The CBF in the whole brain, GM, WM, MFG.L (orbital part), MTG.L, and Tha.R in the patients was lower than that in the HCs, while the CBF in the IFG.L (triangular part) was higher than that in the HCs. There was a significant correlation between Lp-PLA2 levels and CBF in the whole brain and GM, but not in the patients with WM. In addition, CBF of frontal lobe and thalamus was significantly associated with attention and executive function in patients.

Hypertension is the strongest risk factor of WMHs, which has been widely reported in previous cross-sectional and prospective studies (29). We also observed that hypertension was independently associated with WMHs (*B* = 1.779, *p* = 0.001), which was consistent with previous studies. Hypertension can effect CBF through numerous mechanisms including arteriosclerosis, cerebral autoregulation impairment, vascular collagenosis, and blood–brain barrier disruption (30). These pathological changes manifest as WMHs on MRI. We also noticed that Lp-PLA2 was an independent risk factor for WMHs. Lp-PLA2, a macrophage-derived proinflammatory enzyme that participates in the metabolism of low-density lipoprotein (LDL) cholesterol and HDL, contributes to vulnerable atherosclerotic plaques and is associated with cardiovascular and cerebrovascular stroke and dementia (15, 31, 32). There was a significant difference in Lp-PLA2 levels, not hsCRP levels, between patients with WMHs and HCs. Our findings were consistent with several previous studies (15, 21, 33) in which vascular inflammation played a stronger and more consistent role in WMHs than systemic inflammation. However, observations from another study showed that Lp-PLA2 levels in patients with severe WMHs were significantly lower than those with mild or moderate WMHs (20), which suggested anti-inflammatory functions of Lp-PLA2 under certain circumstances (34). The proposal that LDL-associated Lp-PLA2 was atherogenic, while HDL-associated enzymes had the opposite effect and that different detection methods were used could partly explain the inconsistent results (32). A longitudinal investigation demonstrated that higher or elevated levels of hsCRP at midlife predicted greater progression and structural abnormalities of WMHs at late life (19, 35). These results suggested that increased and prolonged systemic inflammation could promote the development of WMHs to a certain extent instead of transient inflammation.

There was evidence of a negative correlation between Lp-PLA2 levels and whole-brain CBF in patients with WMHs. Regional differences were also observed, whereby Lp-PLA2 appeared to be preferentially related to GM CBF but not WM CBF. The arterial transit times of PCASL in the WM were significantly longer than those in GM; thus, the sensitivity of PCASL MRI in the WM is lower than in GM, which may partly explain the discrepant results between GM and WM. In addition, the blood supply in GM is much higher than that in WM because the terminal branches of the anterior, middle, and posterior arteries of the brain are mostly distributed in GM and anastomosed vascular networks communicate with each other. Thus, the GM is more vulnerable to vascular inflammation. Evidence suggests that vascular inflammation could induce oxidative stress and inflammatory responses, lead to vascular endothelial dysfunction (36), blood–brain barrier breakage (37), atherosclerotic plaque formation, lumen stenosis, and hemodynamic impairment, eventually causing WMHs. Therefore, Lp-PLA2 inhibitors might retard hypoperfusion and progression of WMHs. In regard to clinical applications of Lp-PLA2 inhibitors, although some studies found Lp-PLA2 to be independently associated with AD and increased the risk of AD (38), clinical trials with darapladib, the most advanced Lp-PLA2 inhibitor, failed in 2 phase III trials (39, 40). Whether vascular inflammation is causally related to pathogenesis of WMHs or merely a secondary mechanism of WMHs remains unclear and inconclusive. We merely examined inflammatory markers at a single time point. The potential role of inflammatory markers is worth further exploration and prospective studies detecting various inflammatory markers along with progression of WMHs at multiple time points will be needed.

The CBF ensures adequate delivery of oxygen, energy, and nutrients as well as removal of metabolites, which is essential to maintain healthy brain function. In agreement with previous studies (41), the CBF values in the patients were significantly lower than those in the HCs in the whole brain, GM, and WM, with a few exceptions (42). Changes in perfusion are observed not only in the global range, but also in specific regions. Our observations showed the existence of regionally-specific hypoperfusion areas in the orbital frontal cortex, temporal cortex, and thalamus, which was consistent with some prior study (43–45) but not with other studies (46). Moreover, previous studies confirmed a negative association between volume of WMHs and CBF values in the orbital frontal region (47). Of note, the results showed that both the hypoperfusion and hyperperfusion areas existed and we found that hyperperfusion in the IFG.L (triangular part) region was presented in WMHs, which was possibly attributable to neuronal adaptive responses and compensatory mechanisms in very early pathological processes to maintain cognitive function that actually reflect dysregulation of the neurovascular unit (NVU) (48). The hypoperfusion and hyperperfusion areas may simultaneously involve different brain regions and the asymmetrical results in this study may be due to the dominant left hemisphere being more often associated with various advanced cognitive functions (49). Prior findings provided a novel hypothesis of a possible inverted U pattern of the CBF across the different stages of degenerative disease (46). Thus, the discrepant results could be explained further in this way because we did not group the patients with WMHs based on severity and course of disease. In addition, the different scanning protocols, parameters, and post-processing analysis methods could also have led to different results.

Evidence from previous studies revealed that perfusion disturbances may predate microstructural alterations and visible lesions (2, 50), which strongly supported an etiologic contribution. Inconsistent findings were found with regard to longitudinal studies comparing the chronological order of occurrence of hypoperfusion and microstructural abnormalities. Some study discovered that the CBF predicted the development of new WMHs over time and was more strongly associated with cognitive dysfunction (51, 52) and the reverse has also been shown (53, 54). The biological significance of these observations remains a matter of debate. The NVU may regulate CBF through contraction and dilation of vessels and influence angiogenesis, which affects the integrity of WM tracts and finally leads to cognitive decline.

Perfusion is increasingly considered to play a pivotal role in WMHs and stable CBF is fundamental to maintaining brain function. We performed a voxel wise analysis to further probe the relationship between CBF in spatial areas and cognitive deficits. There was significant correlation between the CBF of MFG.L (orbital part), Tha.R, IFG.L (triangular part) and attention, execution function. Previous studies have shown that the frontal lobe plays an important role in the default mode network, frontoparietal network, and dorsal attention network (55). The role of the frontal lobe and thalamus in attention/executive function has been well established and prior longitudinal study assessing ASL perfusion in 136 aging adults found that greater baseline perfusion in the right thalamus and left dorsolateral prefrontal cortex was linked to better executive function (24). Disruption of the interaction between frontal-subcortical circuits, the basal ganglia, and the thalamus leads to disconnection syndrome and mediates brain cognitive functions (56). Accumulating evidence has showed that the thalamofrontal circuit is vital for attention/executive function (25, 57, 58). More strikingly, recent clinical studies revealed that higher CBF values in the frontal cortex corresponded with worse cognitive performance (59). Meanwhile, longitudinal study showed that CBF changes were not related to neurocognitive changes (60). Despite controversial observations, several longitudinal studies reported that frontal perfusion predicted later cognitive performance at follow-up (25, 61). In the context of this prior study, another follow-up study validated that individuals with mild cognitive impairment who later converted to dementia had lower baseline perfusion in the middle frontal cortices than non-converters (24).

Our findings support the use of CBF as a potential marker of WMHs and vascular cognitive impairment. The current findings provide novel insight into interventions targeting of CBF through blood pressure management, diet, and exercise (62, 63). The influence and optimal degree of blood pressure management on CBF has been controversial. Although findings suggest that antihypertensive treatment can repress the progression of WMHs volume over time (64, 65), there was weak evidence from other follow-up studies that found no CBF changes or even increased CBF after antihypertensive use (62, 66). However, some aggressive blood pressure management would impair cerebral autoregulation. Thus, CBF is a relevant clinical target for CBF interventions that prevent future growth of WMHs and the subsequent development of cognitive impairment.

This study had several limitations. First, we focused only on WMHs and neglected other important neuroimaging markers. We did not classify or stratify patients based on the severity of the lesion nor did we measure volume of WMHs. Second, we collected binary values for vascular risk factors and were, thus, unable to verify for associations across a gradient of potential risks such as WMHs and blood pressure. Finally, and most importantly, this study was cross-sectional and had a relatively small sample size, which decreased the statistical power. In the future, larger sample sizes in multicenter and longitudinal research studies are warranted to understand the pathogenesis of WMHs in the context of CSVD.

## Conclusion

In this study, Lp-PLA2 level was an independent risk factor for WMHs, which could influence CBF in the whole brain and GM, while CBF changes in different brain regions may imply a potential role in modulation of cognitive function in different domains. Overall, it is possible that both the inflammation and hypoperfusion mechanisms existed and a pathologic cycle ultimately led to a cascade of damage including lesions and cognitive impairment. Inflammation and hypoperfusion pathways may be crucial targets for prediction and intervention, as they are potentially reversible. Moreover, a vast number of studies have revealed that WMHs are only the tip of the iceberg of pathology of CSVD that the pathophysiological alterations underlying WMHs are a dynamic and highly variable process (67) and that the pathogenesis of WMHs is not well established. This highlights the need for further study to illustrate the underlying mechanism through larger samples and multicenter longitudinal study.

## Data Availability Statement

The raw data supporting the conclusions of this article will be made available by the authors, without undue reservation.

## Ethics Statement

The studies involving human participants were reviewed and approved by the Ethics Committees of the First Affiliated Hospital of Anhui Medical University (Reference no, Quick-PJ 2021-10-16). The participants provided their written informed consent to participate in this study.

## Author Contributions

C-JH, XZ, X-QZ, and Z-WS conceived and designed the experiments. C-JH, XY, WZ, M-ZY, and M-XL performed the experiments. C-JH, XZ, and Z-WS analyzed the data. C-JH and XZ drafted the manuscript. All the authors read and approved the final version of the manuscript.

## Funding

This study was supported by the National Natural Science Foundation of China (81771154), the Key Research and Development Projects of Anhui Province (202104j07020031), the Natural Science Foundation of Anhui Province (1908085QH322), and the Basic and Clinical Cooperative Research Promotion Plan of Anhui Medical University (2020xkjT026).

## Conflict of Interest

The authors declare that the research was conducted in the absence of any commercial or financial relationships that could be construed as a potential conflict of interest.

## Publisher's Note

All claims expressed in this article are solely those of the authors and do not necessarily represent those of their affiliated organizations, or those of the publisher, the editors and the reviewers. Any product that may be evaluated in this article, or claim that may be made by its manufacturer, is not guaranteed or endorsed by the publisher.
