# Supplementary material for: Contribution of Inflammation and Hypoperfusion to White Matter Hyperintensities-Related Cognitive Impairment
Source: Front Neurol. 2022 Jan 4;12:786840. doi: 10.3389/fneur.2021.786840 (PMC8763977; doi:10.3389/fneur.2021.786840)
Supplement: Supplementary file 1 [file Table_1.docx]

**Supplementary Materials**

**Supply Table 1** Relationship between CBF in regions of interest and Neuropsychological Tests in patients with WMHs [*R* (*P* value)].

|  | MFG.L  (orbital part) | MTG.L | Tha.R | IFG.L  (triangular part) |
| --- | --- | --- | --- | --- |
| CAMCOG-C | **0.414(<0.001)** | 0.018(0.888) | 0.188(0.134) | -0.020(0.873) |
| Orientation | **0.553(<0.001)** | -0.071(0.576) | 0.060(0.633) | -0.041(0.747) |
| Language | **0.360(0.003)** | 0.013(0.916) | 0.115(0.362) | 0.091(0.472) |
| Memory | **0.388(0.001)** | -0.005(0.970) | 0.212(0.089) | 0.036(0.778) |
| Attention | 0.201(0.108) | 0.085(0.503) | 0.027(0.833) | **-0.288(0.020)** |
| Execution | **0.444(<0.001)** | -0.150(0.232) | 0.097(0.444) | -0.193(0.124) |
| Calculation | **0.310(0.012)** | -0.045(0.721) | -0.037(0.773) | -0.064(0.610) |
| Abstraction | 0.119(0.346) | 0.074(0.559) | 0.058(0.646) | -0.056(0.657) |
| Perception | 0.198(0.115) | 0.139(0.269) | 0.107(0.396) | 0.037(0.767) |
| MMSE | **0.413(<0.001)** | -0.129(0.305) | 0.162(0.196) | -0.126(0.317) |
| MOCA | **0.367(0.003)** | -0.115(0.360) | 0.051(0.686) | -0.039(0.757) |
| SCWT A | **-0.320(0.009)** | -0.124(0.325) | -0.154(0.219) | 0.161(0.201) |
| SCWT B | **-0.331(0.007)** | -0.031(0.804) | -0.178(0.156) | 0.104(0.409) |
| SCWT C | **-0.262(0.035)** | -0.071(0.576) | **-0.426 (<0.001)** | 0.104(0.409) |
| TMT-A | **-0.437(<0.001)** | 0.074(0.559) | -0.137(0.278) | 0.002(0.990) |
| TMT-B | **-0.431(<0.001)** | 0.099(0.431) | -0.039(0.760) | 0.054(0.670) |

CAMCOG-C, Cambridge Cognitive Examination–Chinese Version; MMSE, Mini-Mental State Examination; MOCA, Montreal Cognitive Assessment; SCWT, Stroop’s color word test; TMT, Trail Making Test; MFG.L (orbital part), left orbital medial frontal gyrus; MTG.L, left middle temporal gyrus; Tha.R, right thalamus; IFG.L (triangular part), left triangular inferior frontal gyrus. *R*, correlation coefficient; Significant differences are indicated in bold.

**Supply Figure 1**| Correlation between CBF in regions of interest and neuropsychological test results. (**A**) Positive correlation between MFG.L (orbital part) CBF values and orientation based on CAMCOG-C scores. (**B**) Positive correlation between MFG.L (orbital part) CBF and language based on CAMCOG-C scores. (**C**) Positive correlation between MFG.L (orbital part) CBF and memory based on CAMCOG-C scores. (**D**) Positive correlation between MFG.L (orbital part) CBF and calculation based on CAMCOG-C scores. (**E**) Positive correlation between MFG.L (orbital part) CBF and MMSE scores. **(F)** Positive correlation between MFG.L (orbital part) CBF and MOCA scores. **(G)** Negative correlation between MFG.L (orbital part) CBF and SCWT-A scores. **(H)** Negative correlation between MFG.L (orbital part) CBF and SCWT-B scores. **(I)** Negative correlation between MFG.L (orbital part) CBF and SCWT-C scores. CBF, cerebral blood flow; MFG.L (orbital part), left orbital medial frontal gyrus; CAMCOG-C, Cambridge Cognitive Examination–Chinese Version; MMSE, Mini-Mental State Examination; MOCA, Montreal Cognitive Assessment; SCWT, Stroop’s color word test.
