# Supplementary figures and images for: Contribution of Inflammation and Hypoperfusion to White Matter Hyperintensities-Related Cognitive Impairment
Source: Front Neurol. 2022 Jan 4;12:786840. doi: 10.3389/fneur.2021.786840 (PMC8763977; doi:10.3389/fneur.2021.786840)

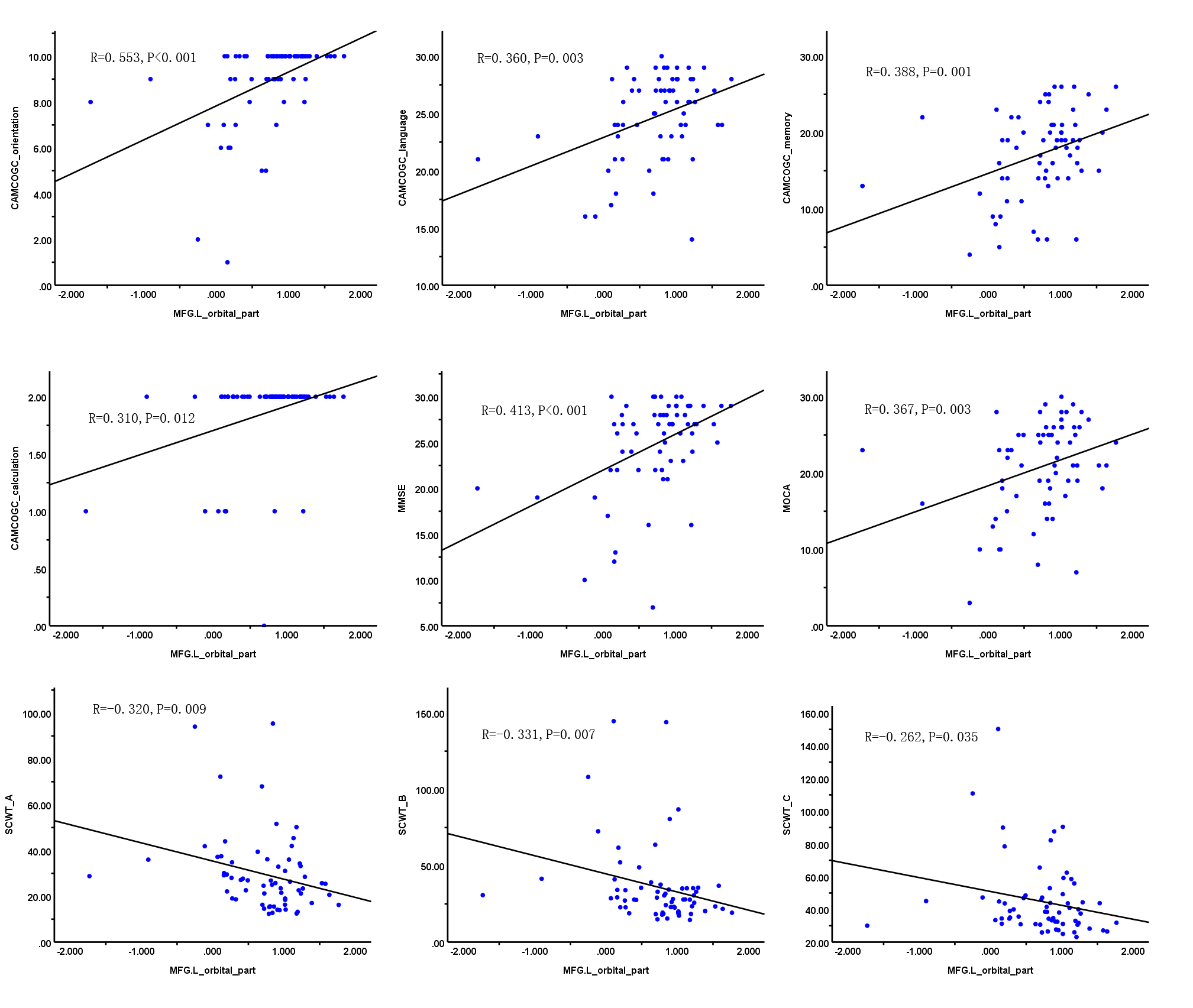

Supplement: Supplementary file 2 [file Image_1.TIF]
